# Bilingual Brains Learn to Use L2 Alliterations Covertly like Poets: Brain ERP Evidence

**DOI:** 10.3389/fpsyg.2021.691846

**Published:** 2021-09-16

**Authors:** Siqin Yang, Xiaochen Zhang, Minghu Jiang

**Affiliations:** ^1^Center for Psychology and Cognitive Science, Tsinghua University, Beijing, China; ^2^Shanghai Mental Health Center, Shanghai Jiao Tong University School of Medicine, Shanghai, China

**Keywords:** bilingual, second-language acquisition, native-language processing, covert translation, priming, alliteration, event-related potential (ERP), N400

## Abstract

Bilinguals were documented to access their native or first language (L1) during comprehension of their second languages (L2). However, it is uncertain whether they can access L2 when reading their first language. This study used the event-related potential (ERP) technique to demonstrate the implicit and unconscious access to English words when Chinese–English bilinguals read words in Chinese, their native language. The participants were asked to judge whether the Chinese words presented in pairs were semantically related or not, meanwhile unconscious of the occasional alliteration (repetition of the first phoneme) if the Chinese words were translated into English. While the concealed prime in English translations failed to affect the reaction time, the alliteration significantly modulated N400 among advanced English learners, especially for semantically unrelated word pairs. Critically, this modulation effect was discrepant between bilinguals with high-level and normal-level English proficiency. These results indicate that L2 activation is an unconscious correlate of native-language processing depending on L2 proficiency.

## Introduction

Connections between bilingual mental lexicons are crucial in bilingual research. Both cognitive scientists and neuroscientists have shown great interest in how individuals store and access words from different languages (Alvarez et al., 2003; Grant et al., 2015). Although some previous evidence support that L2 processing is inevitably accompanied by implicit L1 activations (Thierry and Wu, 2004, 2007; Wu and Thierry, 2010, 2012b), it remains unclear whether L1 processing may also trigger implicit L2 activations. The answer to this question may provide critical insights to the current understandings of mutual connectivity between mental lexicons in bilinguals.

In regard to lexical access, previous research mainly suggested four bilingual models, namely, the Bilingual Model of Lexical Access (BIMOLA) (Grosjean, 1988; Spivey and Marian, 1999), the Revised Hierarchical Model (RHM) (Kroll and Stewart, 1994), the Bilingual Interactive Activation Plus (BIA+) model (Dijkstra and van Heuven, 2002), and the Developmental Bilingual Interactive-Activation (BIA-d) model (Grainger et al., 2010; Legault et al., 2019).

Both the BIMOLA and the BIA+ models focus on the relationship between target and non-target languages. The BIMOLA assumed that bilinguals have two language networks; each has its features, phonemes, words, etc. When bilinguals use one of these languages, they will inevitably suffer from interference from the other languages. In the monolingual mode, the activation level of the network of the language in use is considerably high, and the activation level of other language networks is low. However, in the bilingual mode, multiple language networks are activated at the same time with different degrees depending on proficiency.

The BIA+ model is an upgraded version of the BIA model (Grainger and Dijkstra, 1992; van Heuven et al., 1998). Similar to the BIMOLA, the original BIA model assumes that features, letters, vocabulary, and language constitute a vertical hierarchical relationship within the language recognition system. The input of linguistic information activates all levels from bottom to top. The BIA+ model adds phonetic and semantic representations to the language recognition system. Both BIA and BIA+ models assume that there is a non-selective activation relationship and a lateral inhibition relationship between the target language and the non-target language. The target and non-target languages are first activated in parallel. After the target language is recognized, the non-target language is suppressed. In general, the main difference between the BIMOLA and the BIA+ model is whether the non-target language is completely suppressed.

Proficiency is considered a critical factor affecting lexical access for both the RHM and the BIA-d. The RHM assumes that the lexical system has two levels: a conceptual level and a lexical level. As L1 and L2 store morphologies separately but share concepts, both activations from L1 to L2 and L2 to L1 are possible. Furthermore, it predicts that bilinguals with high proficiency in L2 engage in “direct conceptual” links between semantic and lexical representations, and therefore, they would process L2 words without influences from L1. On the contrary, low proficient bilinguals engage in the “lexical association” link and show stronger cross-language effects.

As to the BIA-d model, it mainly reports how the proficiency of L2 affects the L1 activation during L2 processing. This model claims that during early learning stages, instead of connecting the shared conceptual store directly, semantic processing of L2 will be first processed through the L1. However, during later stages of L2 learning, individuals will inhibit L1 connections for facilitating direct connections to the conceptual store. Nonetheless, whether the activation of L2 could be affected by the level of itself (L2) proficiency or not was still unknown.

The speculation that bilinguals have unified and shared semantic concepts for understanding different languages (Kroll and Stewart, 1994) is supported by behavioral studies, event-related potential (ERP)studies (Guo et al., 2012), and functional neuroimaging studies (Xue et al., 2004; Rodriguez-Fornells et al., 2009). In addition, studies have shown that during L2 processing, bilinguals activate related information of L1 either consciously or unconsciously (Wu and Thierry, 2012b; Ma and Ai, 2018). These studies were in accordance with the BIMOLA, in that when individuals process L2 words, they automatically retrieve semantically related L1 words (Thierry and Wu, 2007; Martin et al., 2009; Costa et al., 2017). For instance, when advanced Chinese-English bilinguals were asked to determine the semantic relevance of English word pairs (e.g., train/ham, 火车/火腿 in Chinese), they unconsciously perceived the repeated character in Chinese translation (e.g., 火). Similar results have also been seen in Korean-English bilinguals (Mishra and Singh, 2016) and English-Spanish bilinguals (Degani and Tokowicz, 2013).

A phoneme may be one of the most sensitive elements in bilingual lexical retrieval (Shook and Marian, 2016; Wen et al., 2018). For instance, when advanced Chinese-English bilinguals were asked to determine the semantic relevance of English word pairs with first characters sharing pronunciation but possessing different morphologies in L1 translation (e.g., experience/surprise, 经验/惊讶 in Chinese, /jing1-yan4/, /jing1-ya4/ in Pinyin) and English word pairs with first characters sharing morphology but possessing different pronunciations (e.g., accounting/meetings, 会计/会议 in Chinese, /kuai4-ji4/, /hui4-yi4/ in Pinyin), their N400 was only affected by the priming effect of pronunciation repetition rather than morphological repetition, suggesting that the L1 they retrieved was in the acoustic form rather than the visual form (Wu and Thierry, 2010; Correia et al., 2014). Moreover, a similar phenomenon was also observed in a semantic matching paradigm (Wang et al., 2017). Results show that when bilinguals read an English word (e.g., fee, 费 in Chinese), only the English word with the same pronunciation in Chinese was activated (e.g., lung, 肺/fei4/ in Chinese).

Since L1 words are activated while bilinguals read L2 words, it remains unclear whether L2 words will be activated while they are reading L1 words. Unlike the L1 activation in L2 processing, the L2 activation in L1 processing is much less investigated and remains ambiguous (Caramazza and Brones, 1979; Gerard and Scarborough, 1989; Jared and Kroll, 2001; Rodriguez-Fornells et al., 2002; van Hell and Dijkstra, 2002; Martin et al., 2009). The models of bilingual access like the BIMOLA and the BIA+ model (Dijkstra and van Heuven, 2002) all take the connectionism assumption that different languages are represented in distributed networks consisting of multiple levels, including orthography, phonology, and semantics (Ma et al., 2014; Kroll et al., 2015) with mutual connections. Hence, it could be hypothesized that L2 is activated during L1 processing.

In a study, Khachatryan et al. (2016) asked Dutch-English bilinguals to determine the semantic relevance of Dutch words. Four Dutch stimuli groups were set. In the homograph unrelated group, an inter-lingual homograph between Dutch and English was presented as a prime word, whereas a Dutch word associated with the English meaning of the prime word was presented as target word (e.g., “star” and “zon,” “zon” meaning “sun” in Dutch). In the homograph related group, an inter-lingual homograph was also used as a prime word, and a Dutch word corresponding to the Dutch meaning of the prime word was presented as a target word (“star” and “stijf,” both meaning “numb” in Dutch). The remaining two groups were for control; one is the semantically related group (e.g., “bloem” and “roos,” “bloem” meaning “flower” and “roos” meaning “rose” in Dutch), and the other is the semantically unrelated group (e.g., “herfst” and “hond,” “herfst” meaning “autumn” and “hond” meaning “dog” in Dutch). Results show an N400 reduction indicating the priming effect for the homograph unrelated group, suggesting that the covert manipulation of L2 (here, English) affects L1 processing. This study claimed that while bilinguals were comprehending L1 words, L2 words were also implicitly activated, supportive of the BIMOLA rather than the BIA+ model.

Dutch and English are both Indo-European languages and share lots of inter-lingual homographs that may be close in the bilingual mental lexicon. Consider Chinese-English bilinguals, since their L1 and L2 belong to different language families, whether L2 is activated during L1 processing remains unclear. This study adopted the implicit priming paradigm as previous studies to further investigate whether the L1 processing could be affected by the possible priming effect of alliteration concealed in L2. A previous study (Thierry and Wu, 2007) set four types of English word pairs (the first word was used as the priming word and the second as the target word) as conditions, semantically related word pairs that shared the first character when translated into Mandarin Chinese (e.g., post and mail, meaning 邮政 and 邮件 in Chinese, S+P+ English pairs), semantically related word pairs that shared no character when translated into Mandarin Chinese (e.g., wife and husband, meaning 妻子 and 丈夫 in Chinese, S+P- English pairs), semantically unrelated word pairs that shared the first character when translated into Mandarin Chinese (e.g., train and ham, meaning 火车 and 火腿 in Chinese, S-P+ English pairs), and semantically unrelated word pairs that shared no character when translated into Mandarin Chinese (e.g., table and apple, meaning 桌子 and 苹果 in Chinese, S-P- English pairs). There were two types of primes, semantic prime and character prime. Results revealed that bilinguals perceived the semantic prime consciously and perceived the character prime unconsciously.

Similarly, this study also adopted the implicit priming paradigm with four types of Chinese word pairs as conditions, semantically related word pairs that shared an alliteration when translated into English (e.g., 公主 and 王子, meaning princess and prince in English, S+P+ Chinese pairs, S+ means semantically related and P+ means the first phoneme repeated), semantically related word pairs that did not share any alliteration when translated into English (e.g., 报纸 and 记者, meaning newspaper and reporter in English, S+P- Chinese pairs, S+ means semantically related and P- means the first phoneme not repeated), semantically unrelated word pairs that shared an alliteration when translated into English (e.g., 博士 and 美元 meaning doctor and dollar in English, S-P+ Chinese pairs, S- means semantically unrelated and P+ means the first phoneme repeated), and semantically unrelated word pairs that did not share any alliteration when translated into English (e.g., 沙滩 and 卡片, meaning beach and card in English, S-P- Chinese pairs, S- means semantically unrelated and P- means the first phoneme not repeated). If the L1 processing was affected by implicit L2 activation as the BIMOLA assumed, we would observe a priming effect in conditions with alliterations in English translations.

The ERP was considered as an ideal methodology to observe how individual brains respond to linguistic information. This study adopted the ERP technique to investigate the activation of L2 during L1 processing. For behavioral performances, previous studies revealed that the RT to the target word of semantically related word pairs was faster than that of the semantically unrelated word pairs due to the semantic priming (Thierry and Wu, 2007). However, whether there was a priming effect for word pairs with implicit alliterations in L2 was still unclear. Since there was no significant difference in RT between L2 target words with and without implicit L1 primes (Thierry and Wu, 2004, 2007; Wu and Thierry, 2010), we predicted that the repetitive prime in L2 may not cause a difference in RT at the behavioral level.

For ERPs results, we focused on the N400 component, a negative deflection peaking around 400 ms post-stimulus onset, which was not only sensitive to semantic relatedness but also to sound repetition (Liu et al., 2003; Kutas and Federmeier, 2011; Brouwer and Crocker, 2017). Therefore, the N400 was regarded as an indicator in this study for both explicit semantic primes and implicit alliterative primes. Previous studies established that the target words of semantically unrelated word pairs elicited a clear N400 and that semantically related prime words before target words reduced N400 amplitude (Thierry and Wu, 2007; Delogu et al., 2019). In addition, a word with a repetitive prime was reported to trigger a reduced N400 amplitude than that without a repetitive prime (Koyama et al., 1992; Delogu et al., 2019). The BIMOLA claimed that L2 was not completely suppressed during L1 processing, and thus, the implicitly alliterative prime in L2 may cause a reduction of N400 amplitude. Considering the RHM again, since the lexical connection from L1 to L2 is not strong, we predicted that the priming effect induced by implicitly alliterative primes in L2 may be weaker than the priming effect induced by explicitly semantic primes.

For both the RHM and the BIA-d models, proficiency seems to be a critical factor affecting lexical access for bilinguals (Kroll and Stewart, 1994). Whether the possible L2 activation could be affected by L2 proficiency was still unclear. This study recruited two groups with different L2 proficiency, high-level and normal-level bilinguals, to explore whether the implicit activation of L2 would be affected by L2 proficiency.

## Methods

### Participants

Two groups of Chinese-English bilinguals participated in the experiment, 24 high-level bilinguals (mean age = 22.25 years, range = 19–26 years old, 12 females) and 24 normal-level bilinguals (mean age = 22.75 years, range = 19–26 years old, 12 females), respectively. All participants were university students from Mainland China. They all started to study English no earlier than 10 years of age and thus could be regarded as late learners of English. The classification of the participants was based on English proficiency. High-level bilinguals met at least one of the two criteria: the score of the International English Language Test (IELTS) was 7 or above, or the score of the Test of English as a Foreign Language Internet-Based Test (TOEFL-iBT) was 105 or above. Normal-level bilinguals met at least one of the following two criteria: the IELTS score was between 5.5 and 6.5, or the TOEFL-iBT score was between 85 and 105. None of them had residence experience in an English-speaking country before. They took TOFEL and IELTS to make preparations for studying abroad in an English-speaking country in the future. The family members of the participants were all from Mainland China, and no participant had living experience in an English-speaking home environment. All the participants had a bachelor's or above degree. High-level bilinguals majored in English at university, whereas normal-level bilinguals did not. Hence, high-level bilinguals conceivably may have more contact with English and more chances to translate between English and Chinese. These factors were not quantitively measured or controlled between the two groups in this study. All the participants had normal vision or corrected vision. None had neurological or psychological diseases. All the participants were righthanded as tested by the Edinburgh hand test. No participant participated in the evaluation of the words used as stimuli in the experiment. No participant had perceived the hidden alliteration in the English translations of the words before the experiment was finished, confirmed by a questionnaire after the experiment. All participants were paid for their time and gave written informed consent to the experimental protocol approved by the local Ethics Committee at Tsinghua University.

### Materials and Design

There were 84 Chinese word pairs used in the experiment, divided into 4 groups, namely, S+P+ Chinese pairs, S+P- Chinese pairs, S-P+ Chinese pairs, and S-P- Chinese pairs (S+ means semantically related, P+ means the first phoneme of English translation repeated, S- means semantically unrelated, and P- means the first phoneme of English translation not repeated). A typical set of stimuli is given in Table 1. Each group contained 21 Chinese word pairs (all given in the Supplementary Material, as well as their English translations).

**Table 1:** Experimental design and stimuli examples.

**English alliteration**	Semantic relatedness (explicit factor)	
**(Implicit factor)**	**Semantic related (S+)**	**Semantic unrelated (S-)**
Repetition(P+)	公主—王子	博士—美元
	Princess—Prince	Doctor—Dollar
	SRC: 4.10(±0.39)	SRC: 1.50(±0.32)
	SRE: 3.80(±0.25)	SRE: 2.00(±0.38)
Repetition(P-)	报纸—记者	沙滩—卡片
	Newspaper—Reporter	Beach—Card
	SRC: 4.50(±0.32)	SRC: 1.5(±0.26)
	SRE: 3.85(±0.63)	SRE: 1.85(±0.19)

The semantic relevance and the word frequency of the stimuli were controlled between different conditions. The semantic relevance of the word pairs and the semantic relevance of the English translations of the word pairs were evaluated with a 5-point Likert scale by a separately recruited group of 20 participants. Two ANOVAs with semantic relatedness (related and unrelated) and alliteration in L2 (alliterative and non-alliterative) as two factors were conducted, one for the semantic relevance of Chinese word pairs and the other for the semantic relevance of English translations. As to the semantic relevance of Chinese word pairs, the semantic relatedness × alliteration in L2 interaction was significant [*F*_(1,80)_ = 4.164, *p* = 0.045]. Furthermore, *t*-tests showed that the semantic relevance of S+P+ word pairs was significantly higher than the S-P+ word pairs [*t*(40) = 23.452, *p* < 0.001], the semantic relevance of S+P- word pairs was significantly higher than the S-P- word pairs [*t*(40) = 31.725, *p* < 0.001], the semantic relevance of S-P+ word pairs was similar to the S-P- word pairs [*t*(40) = 0.318, *p* = 0.752], but the semantic relevance of S+P- word pairs was significantly higher than the S+P+ word pairs [*t*(40) = 2.382, *p* = 0.022]. A slightly higher semantic relevance of S+P- word pairs than S+P+ word pairs would only reduce the possibility for the priming effect of alliteration on the N400 to be observed under semantically relevant conditions and thus would not impair the validity of the experimental design of this study. As to the semantic relevance of English translations, the main effect of semantic relatedness was significant [*F*_(1,80)_ = 427.757, *p* < 0.001]. Neither the main effect of alliteration in L2 nor the semantic relatedness × alliteration in L2 interaction was significant. An analysis of variance (ANOVA) with semantic relatedness (related and unrelated) and alliteration in L2 (alliterative and non-alliterative) as two factors was conducted for the word frequency. No main effect or interaction was significant.

To ensure the consistency between Chinese words and their English translation, another separately recruited group of 10 participants was asked to determine whether the first English translation that popped into their minds when they saw a Chinese word was the same as the English translation adopted in this study. Therefore, each Chinese word had a score on the translation consistency represented by how many people would first think of the adopted English translation after viewing the Chinese word. An ANOVA with semantic relatedness (related and unrelated) and alliteration in L2 (alliterative and non-alliterative) as two factors was conducted for the translation consistency score. No main effect or interaction was significant.

As alliteration is the key prime in the present experiment, the average number of syllables in English translations was reported, 1.738 (SD = 0.912) in S+P+ Chinese pairs, 1.976 (SD = 0.869) in S+P- Chinese pairs, 1.833 (SD = 0.794) in S-P+ Chinese pairs, and 1.714 (SD = 0.774) in S-P- Chinese pairs. An ANOVA with semantic relatedness (related and unrelated) and alliteration in L2 (alliterative and non-alliterative) as two factors was conducted for the number of syllables of English translation words. No main effect or interaction was significant.

Owing to the limited number of Chinese word pairs satisfying the requirements of the experimental protocol, 84 pairs were repeated three times in the experiment, forming a total of 252 trials to ensure the number of measurements. These stimuli were presented pseudorandomly.

### Procedure

Participants were seated in a comfortable chair in a quiet room with a distance of 80 cm to the computer screen. All stimuli were in white and were presented on a gray background on the screen, under control of the E-Prime software (2.0, Psychology Software Tools). The font was Song typeface with a size of 34. Participants were informed to determine quickly whether the Chinese word pairs presented on the screen are semantically related or not and respond by pressing a button on a joystick. The responding hand was counterbalanced across participants. The whole experiment was divided into four blocks. The first block was a practice block, where 20 pairs of Chinese words, 10 semantically related and 10 unrelated, were presented to the participants. The next three blocks were formal blocks. In each formal block, 84 word pairs, 21 for each of the four conditions, were presented in the pseudorandom order. Every formal block shared the same stimuli. Data analyses were based only on formal blocks.

The process of a trial was as follows: the fixation appeared in the center of the screen for 200 ms to remind participants of focusing attention; then, the first Chinese word appeared in the center of the screen for 500 ms; after that, the first word disappeared left with an empty screen for a random period of time (500, 600, or 700 ms); finally, the second word appeared on the screen and did not disappear until participants responded; after responses, a blank screen appeared again for a random period of time (200, 300, or 400 ms). Then, the next trial started.

At the end of the experiment, participants were asked in a questionnaire whether they found that some word pairs had the same first syllable when translated into English. None of the participants reported that they had found the hidden conditions of alliteration in English translation during the experiment.

### Electroencephalogram Recording and Pre-processing

A 61 Ag/AgCl electrodes elastic cap with an international 10–20 electrode placement system (Easycap; Brain Products GmbH, Gilching, Germany) was used to record the EEG data from participants. FCz and AFz electrodes were used as the reference electrode and the ground electrode, respectively. Two ocular electrodes were used to measure the vertical and the horizontal electrooculogram (EOG), placed on the lower side of the left eye and the outer side of the right eye, respectively (Picton et al., 2000). The electrode impedance was maintained under 10 kΩ. The EEG data were recorded with the BrainAmpDC amplifier system (Brain Products GmbH) with a bandpass of 0.01–100 Hz, under control of the Brain Vision Recorder software (Brain Products, Munich, Germany). The protocol of the electrophysiological recording resembled our previous study (Zhang et al., 2020).

The EEG data were analyzed with the Brain Vision Analyzer software (Brain Products, Munich, Germany), including re-referencing, EOG correction, filtering, segmentation, baseline correction, artifact rejection, and averaging. The EEG data were first re-referenced to the mathematically-linked mastoids, and then to the average of all EEG channels after the pre-processing. Then, ocular artifacts were corrected with the independent-component-analysis-based procedure embedded in the Brain Vision Analyzer software (Brain Products, Munich, Germany). Next, the EEG data were bandpass filtered offline from 0.1 to 35 Hz. The EEG from 200 ms before the onset of the target word to 800 ms after the onset was segmented into epochs (200 ms pre-target baseline). Epochs with possible artifacts induced by movements (voltage exceeding ±80 μV) were discarded.

### Data Analyses

We focused on the reaction time and the response accuracy rate for analyses of behavioral performances. The reaction time was the median time one took to respond to target words. The response accuracy rate was the proportion of trials with correct responses. For ERP analyses, we focused on the N400 component, which was widely recognized as an index of semantic relevance and priming (Lau et al., 2013). In this study, the N400 component was investigated *via* the averaged ERP recorded at the electrodes FCz, FC1, FC2, Cz, C1, C2, C3, and C4; this electrode cluster was determined by visual inspection of the grand-averaged scalp topographies of the N400. The analysis window was 230–430 ms, determined by visual inspection to include most of the negative deflection that appeared around 400 ms at the electrode cluster. The selected electrode cluster and the time window were similar as reported in previous studies (Thierry and Wu, 2007; Wu and Thierry, 2010; Liang and Chen, 2014). The N400 amplitude reported in this study was the mean amplitude of the ERP in the analysis window at the electrode cluster.

The data were analyzed with three-way repeated-measures ANOVA with semantic relatedness (related and unrelated), alliteration in English (alliterative and non-alliterative) as within-participant factors, and proficiency (high-level and normal-level) as a between-participant factor. Significant interaction effects were followed by *post-hoc* simple effect comparisons by two-way repeated-measures ANOVA and paired *t*-tests.

## Results

### Behavioral Data

**Figure 1:**
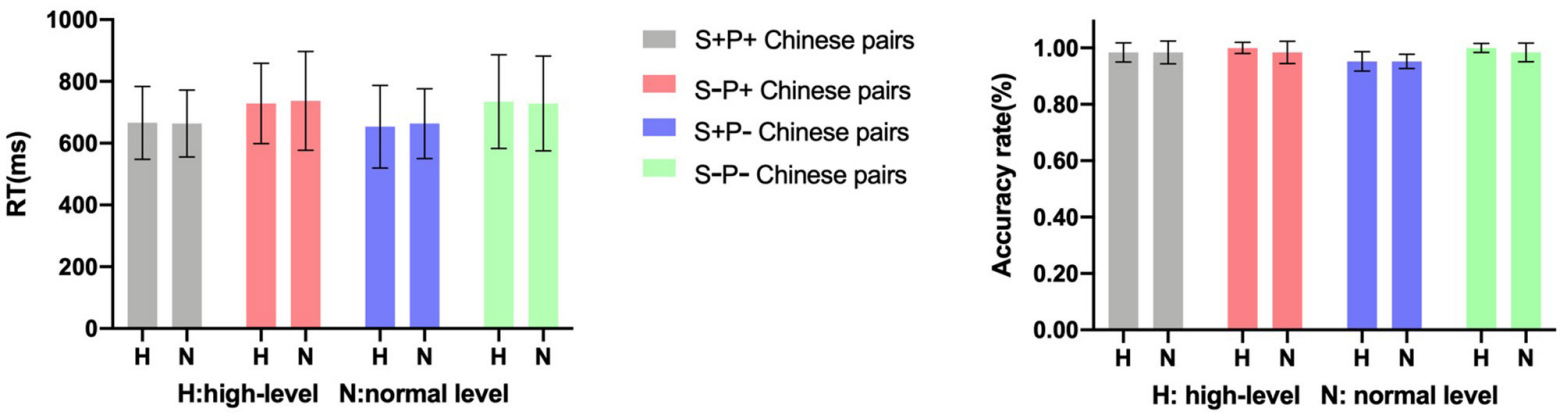
Mean RT and accuracy rates for the two groups in four factors (error bars indicate standard error).

The reaction time (RT) was relative to the onset of the second word. Both the RT and the accuracy rate of the two groups are given in Figure 1. The mean accuracy was over 95% for each condition, 96.73% (SD = 0.533%) in S+P+ Chinese pairs, 95.57% (SD = 0.436%) in S+P- Chinese pairs, 98.15% (SD = 0.452%) in S-P+ Chinese pairs, and 97.85% (SD = 0.400%) in S-P- Chinese pairs. The high accuracy ensures the validity of the data.

With respect to RT, a three-way repeated-measures ANOVA was performed including semantic relatedness (related and unrelated) and alliteration in L2 (alliterative and non-alliterative) as within-participant factors and proficiency (high level and normal level) as a between-participant factor. We observed a significant main effect of relatedness [*F*_(1,46)_ = 87.019, *p* < 0.001, η^2^ = 0.654]; the RT of the semantically related word pairs was shorter than that of the semantically unrelated word pairs (*p* < 0.001), consistent with previous research (Thierry and Wu, 2007). No main effect of alliteration in L2 [*F*_(1,46)_ = 0.007, *p* = 0.931, η^2^ < 0.001], proficiency [*F*_(1,46)_ = 0.108, *p* = 0.744, η^2^ = 0.002], or interactions of semantic relatedness × alliteration in L2 [*F*_(1,46)_ = 2.484, *p* = 0.122, η^2^ = 0.051], alliteration in L2 × proficiency [*F*_(1,46)_ = 0.019, *p* = 0.892, η^2^ < 0.001], semantic relatedness × proficiency [*F*_(1,46)_ = 0.075, *p* = 0.785, η^2^ = 0.002], or semantic relatedness × alliteration in L2 × proficiency [*F*_(1,46)_ = 0.003, *p* = 0.959, η^2^ < 0.001] was observed.

These results indicate that participants with both high-level and normal-level proficiencies were sensitive to semantic relatedness but insensitive to the alliteration in L2. To summarize, there is no behavioral evidence in favor of any unconscious activation from L1 to L2 or interaction with L2 proficiency.

### Electrophysiological Data

**Figure 2:**
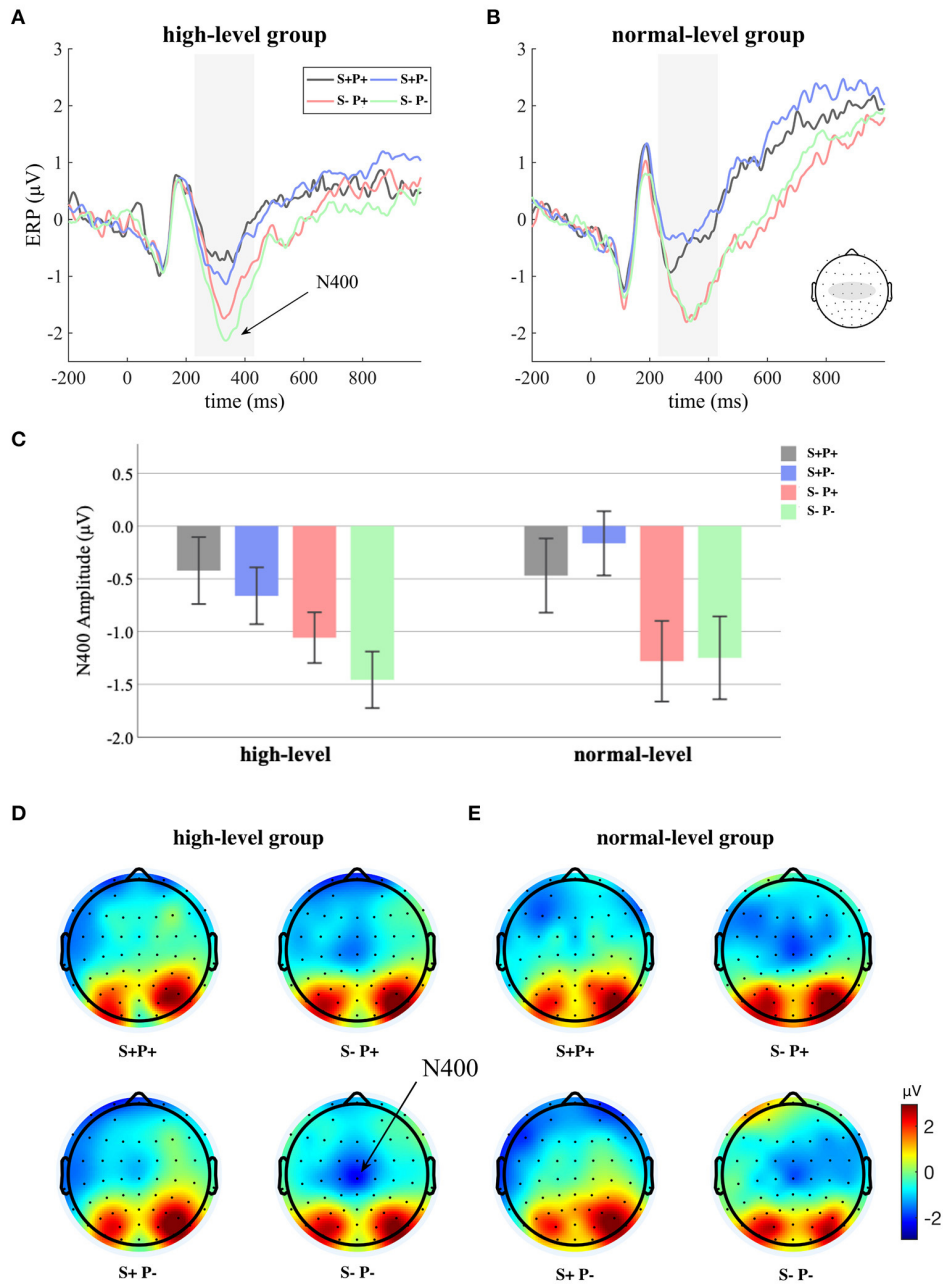
**(A,B)** The grand-averaged ERP waves; **(C)** the N400 amplitude (error bars indicate standard error); **(D,E)** the scalp topographies of N400.

We used the ERP technique to reveal the covert procedure related to L2 during L1 processing. The grand-averaged ERP waves, the N400 amplitude, and the scalp topographies of N400 are displayed in Figure 2.

First, a three-way repeated-measures ANOVA was performed with semantic relatedness (related and unrelated) and alliteration in L2 (alliterative and non-alliterative) as within-participant factors and proficiency (high-level and normal-level) as a between-participant factor. We observed a highly significant main effect of semantic relatedness {semantically related > semantically unrelated; [*F*_(1,46)_ = 49.122, *p* < 0.001, η^2^ = 0.516]}, suggesting a reducing effect of semantic primes on N400 amplitude. In addition, we observed an alliteration in L2 × proficiency interaction [*F*_(1,46)_ = 5.995, *p* = 0.018, η^2^ = 0.115], suggesting that English proficiency affected the priming effect induced by the implicit alliteration in L2. No other main effect or interaction was significant.

Then, two repeated-measures ANOVA were performed, one for participants with high-level L2 proficiency and the other for those with normal-level L2 proficiency, with semantic relatedness (related and unrelated) and alliteration in L2 (alliterative and non-alliterative) as within-participant factors. For the high-level group, we observed a significant main effect of semantic relatedness {semantically related > semantically unrelated; [*F*_(1,23)_ = 16.516, *p* < 0.001, η^2^ = 0.418]}, suggesting a priming effect of semantically related words on the N400. We also observed a significant main effect of alliteration in L2 {alliterative > non-alliterative; [*F*_(1,23)_ = 4.697, *p* = 0.041, η^2^ = 0.170]}, suggesting a priming effect of words whose L2 translation had an alliteration. However, for the normal-level group, we only observed a significant main effect of semantic relatedness {semantically related > semantically unrelated; [*F*_(1,23)_ = 35.488, *p* < 0.001, η^2^ = 0.607]}. The main effect of alliteration in L2 was not significant for the normal-level group [*F*_(1,23)_ = 1.581, *p* = 0.221, η^2^ = 0.064]. For both groups, the interaction of the two within-participant factors was insignificant.

Moreover, we conducted four paired *t*-tests to examine whether there was a reducing effect of implicit L2 primes on the N400 amplitude for each group for each semantic condition. Among participants with high-level English proficiency, we saw a significant reducing effect of implicit L2 primes on the N400 amplitude for semantically unrelated word pairs [alliterative > non-alliterative; *t*(23) = 2.167, *p* = 0.041, two-sided]. With the paired *t*-test, we failed to see a significant priming effect of implicit L2 primes on the N400 amplitude for semantically related word pairs [*t*(23) = 1.094, *p* = 0.285, two-sided]. Among participants with normal-level English proficiency, we did not see any effect of implicit L2 primes on the N400 amplitude.

To summarize, we observed a significant effect of implicit L2 priming (i.e., alliteration in L2) on the amplitude of the N400 elicited during L1 processing among participants with high-level L2 proficiency, especially when semantic primes were absent. This priming effect was absent among participants with normal-level L2 proficiency.

## Discussion

The two main purposes of this study were (1) to explore whether bilinguals automatically activated L2 translations while processing the semantics of L1 words, and, if so, (2) to examine whether the unconscious activation of L2 was modulated by the proficiency of L2. For the first purpose, this study adopted an implicit priming paradigm with four types of Chinese word pairs as stimuli. The activation of L2 during L1 processing was measured by testing whether the participants were sensitive to the implicitly alliterative prime in L2 word pairs. For the second purpose, this study recruited bilinguals with different levels of proficiency to participate in the experiment and compared their behavioral and ERPs results. At the behavioral level, we observed a priming effect of semantically related words, represented by a shorter reaction time, but we did not see any priming effect of the implicit alliteration in L2 translations on the reaction time. At the electrophysiological level, we still observed priming effects of semantically related words, represented by smaller N400 amplitude. Importantly, we also saw a priming effect of the alliteration in L2 translations on the N400 amplitude, but only among participants with high-level L2 proficiency. These results suggest that (1) a covert, unconscious translation to L2 during L1 processing is possible; and that (2) this translation is very likely to be modulated by the L2 proficiency. On the whole, the present results seem to be inconsistent with the BIA+ but in accordance with BIMOLA. Three aspects of these results warrant addressing in terms of implicity and proficiency (Illes et al., 1999; Chee et al., 2003; Tan et al., 2003; Frenck-Mestre et al., 2005; Rodriguez-Fornells et al., 2005; Crinion et al., 2006; Lövdén et al., 2013; Liu et al., 2020).

### Implicity

We found that the activation of L2 during L1 seems to be an implicit procedure. The participants did not realize that there were alliterative connections between the English translations of some of the word pairs until told after the experiment. Besides, the implicitly alliterative prime did not induce any priming effect on the RT, either between S+P+ Chinese pairs and S+P- Chinese pairs or between S-P+ Chinese pairs and S-P- Chinese pairs. Noticeably, the N400 amplitude significantly declined if the target word followed a prime word that shared an alliteration in English translation, indicating that this cross-language activation of L2 in L1 processing can occur implicitly and unconsciously. These results fell in line with the well-known viewpoint in brain neuroscience that the brain knows a lot more than we think it does. Besides, this study makes a meaningful contribution to the current literature on the bilingual model by showing human brains can unconsciously activate the corresponding words beyond the language in which these words appear in a monolingual environment, whether in L1 or L2.

An advantage of the experimental design of this study is its attempt to eliminate possible L2 activations from other sources rather than the implicit alliteration in L2 between some word pairs. We did not present words in different languages to participants, thus avoiding the possibility that the participants felt it necessary to translate the words they saw into English. Some previous cross-language studies (Christoffels et al., 2007; Ma and Ai, 2018) presented both L1 and L2 stimuli in the experiment. For example, Ma and Ai (2018) designed a behavioral experiment (1) to examine when L2 learners read L2 words, whether the L1 translation is activated, and, if so, (2) to examine whether the translation would be modulated by the proficiency in the L2. They recruited elementary Chinese-English bilinguals to quickly determine whether word pairs made up of English words and Chinese words are semantically related or not. In their experiment, the prime word was in English (e.g., cup, 杯, /bei1/ in Pinyin), and the target word was four different types of Chinese words (杯, cup, /bei1/; 坏, bad, /huai4/; 悲, sad, /bei1/; 弱, weak, /ruo4/). They found that only the orthographic information rather than the phonological information of L1 words was activated. Meanwhile, this activation is not modulated by L2 proficiency. However, since both languages were present in the experiment, the experiment possibly failed to rule out alternative sources of L2 activation. Therefore, the L1 activation in this study was likely induced deliberately. Moreover, the activation within such research design was not unconscious (Moreno et al., 2002). In contrast to this study ensured an undisturbed L1 environment, and we could safely assume that the L2 activation was implicit.

### Proficiency

In addition, we found that only the high-level group activated the L2 word, supporting that the covert translation from L1 to L2 is probably modulated by L2 proficiency in a pure L1 environment. These results are consistent with cognitive research on bilingualism, which indicates that different languages of a bilingual access a common semantic system (Illes et al., 1999; Chee et al., 2003; Tan et al., 2003; Frenck-Mestre et al., 2005; Rodriguez-Fornells et al., 2005; Crinion et al., 2006; Lövdén et al., 2013; Liu et al., 2020). On another note, the L2 proficiency in bilinguals affects the activation of brain regions related to language processing, especially in parahippocampal gyrus and cingulate gyrus (Nichols and Joanisse, 2016). Furthermore, both the parahippocampal gyrus and cingulate gyrus contributed to the engenderment of the N400 (Silva-Pereyra et al., 2003; O'Hare et al., 2008). Therefore, our finding that the N400 effect of implicitly alliterative priming is modulated by the L2 proficiency seems to be in accordance with previous neuroimaging studies.

In general, our findings are in accordance with the predictions by the BIMOLA, in that in the monolingual environment, the second language is not completely suppressed. Instead, we found that the second language is covertly activated during the processing of the first language. Our findings are not consistent with the BIA+ model, which predicts that the non-target language is completely suppressed. In addition, our discovery of the modulation for L2 proficiency on the covert translation from L1 to L2 among advanced L2 learners demonstrated that the proficiency indeed modulates cross-language activations. These results also supplement the BIA-d model in that the multiple directions of cross-language activations may be modulated by proficiency. Moreover, our finding that bilinguals with a higher L2 proficiency show greater cross-language activations is against the RHM, which predicts that bilinguals with high proficiency in L2 directly link semantic and lexical representations and suppress L1 activation, whereas low proficient bilinguals may utilize the cross-language lexical-association link and presumably show stronger cross-language effects. Further studies are required to reveal possible functional benefits brought by the covert translation from L1 to L2.

There are several limitations of this study. First, owing to the limited number of Chinese word pairs satisfying the requirements of the experimental protocol, 84 pairs were repeated three times in the experiment, forming a total of 252 trials to ensure the number of measurements. While it may reduce the potential effect of the hidden L2 manipulation, this effect was still observed. In the future, we will try to improve the stimulus material for further study. Second, due to the limitations of the experimental environment, we only recruited two groups of bilinguals with different levels as participants. A better practice would have been to recruit a Chinese monolingual group as control as in previous studies (Thierry and Wu, 2007; Wu and Thierry, 2010, 2012a). Fortunately, the most pivotal effects of the previous studies were mostly found in the Chinese-English bilingual group. Based on this research, the present study aims to further explore whether bilinguals have unconscious L2 activations without consciousness when they read their L1. Third, previous studies conducted both listening and viewing experiments and reached consistent results between the two modalities (Thierry and Wu, 2007; Wu and Thierry, 2010). Instead, we presented the Chinese word pairs only in the visual modality but not in the auditory modality. Further verification of the findings reported in this study is required *via* the adopted paradigm with the stimuli presented aurally. Finally, the participants in the high-level English proficiency group all majored in English in university, whereas the participants in the normal-level group did not. This bias probably affects how to interpret the results of this study. Indeed, the high-level group had a higher proficiency in English than the normal-level group. However, they generally had more contact with English and more chances to translate between English and Chinese. Since these factors were not controlled in this study, the possibility cannot be ruled out that it was the more contact with English or more chance to translate between English and Chinese of the high-level group that induced the priming effect of implicit English alliterations.

## Conclusion

To conclude, this study documents an unconscious activation of the L2 during the processing of the L1, suggesting a covert translation from L1 to L2. Notably, this covert cross-language activation occurs only among advanced learners of L2, suggesting that the covert translation from L1 to L2 is probably modulated by L2 proficiency. This study also provides electrophysiological evidence supporting the activation of L2 in a pure L1 environment, bringing new insights to the theoretic models of bilingual lexical access. Future studies may focus on whether the degree of mutual activation between different languages is symmetrical or not, whether the findings of this study could be generalized to languages other than Chinese and English, and whether this mutual activation may appear in other forms in terms of syntax, tense, or context.

## Data Availability Statement

The data supporting the conclusions of this article are available without undue reservation upon request to the correspondence author.

## Ethics Statement

The studies involving human participants were reviewed and approved by the ethics committee of the Department of Psychology at Tsinghua University. The participants provided their written informed consent to participate in this study.

## Author Contributions

SY, XZ, and MJ conceived the study. SY and MJ designed the experiments. MJ provided the place, the instruments, and the software. SY recruited the participants, performed the data collection, organized and preprocessed all the data, and analyzed the behavioral data. SY and XZ analyzed the electrophysiological data. SY wrote the initial version of the manuscript. SY, XZ, and MJ revised the manuscript. All authors contributed to the article and approved the submitted version.

## Funding

This work was supported by the National Natural Science Key Foundation of China, Research on Cognitive Mechanism and Computational Model of Language Understanding (62036001) and the National Social Science Major Foundation of China, Research on Advanced Cognition at the Level of Language, Thought and Culture (15ZDB017).

## Conflict of Interest

The authors declare that the research was conducted in the absence of any commercial or financial relationships that could be construed as a potential conflict of interest.

## Publisher's Note

All claims expressed in this article are solely those of the authors and do not necessarily represent those of their affiliated organizations, or those of the publisher, the editors and the reviewers. Any product that may be evaluated in this article, or claim that may be made by its manufacturer, is not guaranteed or endorsed by the publisher.
